# Cyclic guanosine monophosphate does not inhibit gonadotropin-induced activation of mitogen-activated protein kinase 3/1 in pig cumulus-oocyte complexes

**DOI:** 10.1186/1477-7827-13-1

**Published:** 2015-01-07

**Authors:** Milan Blaha, Lucie Nemcova, Radek Prochazka

**Affiliations:** Laboratory of Developmental Biology, Institute of Animal Physiology and Genetics, Academy of Sciences of the Czech Republic, Rumburska 89, 277 21 Libechov, Czech Republic

**Keywords:** Pig, Oocyte, cGMP, Meiotic resumption, Expansion-related genes, MAPK3/1

## Abstract

**Background:**

Recent results indicate a key role for cyclic guanosine monophosphate (cGMP) in the regulation of oocyte meiotic arrest in preovulatory mammalian follicles. The aim of our study was to determine whether the resumption of oocyte meiosis and expansion of cumulus cells in isolated pig cumulus-oocyte complexes (COCs) can be blocked by a high intracellular concentration of cGMP, and whether this effect is mediated by a cGMP-dependent inhibition of mitogen-activated protein kinase 3/1 (MAPK3/1).

**Methods:**

The COCs were isolated from ovaries of slaughtered gilts and cultured in vitro in M199 supplemented with 5% fetal calf serum. The expression levels of the C-type natriuretic peptide (CNP) precursor (*NPPC*) and its receptor (*NPR2*) mRNAs during the culture of COCs were determined by real-time RT-PCR. To control the intracellular concentration of cGMP in the COCs, the culture medium was further supplemented with CNP or various concentrations of synthetic cGMP analogues; the concentration of cGMP in COCs was then assessed by ELISA. The effect of the drugs on oocyte maturation was assessed after 24 and 44 h of culture by determining nuclear maturation. The expansion of cumulus cells was assessed by light microscopy and the expression of cumulus expansion-related genes by real-time RT-PCR. A possible effect of cGMP on FSH-induced activation of MAPK3/1 was assessed by immunoblotting the COC proteins with phospho-specific and total anti-Erk1/2 antibodies.

**Results:**

The COCs expressed *NPPC* and *NPR2*, the key components of cGMP synthesis, and produced a large amount of cGMP upon stimulation with exogenous CNP, which lead to a significant (P < 0.05) delay in oocyte meiotic resumption. The COCs also responded to cGMP analogues by inhibiting the resumption of oocyte meiosis. The inhibitory effect of cGMP on meiotic resumption was reversed by stimulating the COCs with FSH. However, high concentration of intracellular cGMP was not able to suppress FSH-induced activation of MAPK3/1 in cumulus cells, cumulus expansion and expression of expansion-related genes (P > 0.05).

**Conclusions:**

The findings of this study indicate that high cGMP concentrations inhibit the maturation of pig oocytes in vitro but the inhibitory mechanism does not involve the suppression of MAPK3/1 activation in cumulus cells.

## Background

Mammalian oocytes are held in the meiotic prophase arrest induced by high intracellular concentration of cyclic adenosine monophosphate (cAMP), which is maintained by the activity of an orphan receptor GPR3 [1, 2] and the inhibition of oocyte-specific phospodiesterase 3A (PDE3A) [3, 4]. Mouse PDE3A was found to be inhibited by cyclic guanosine monophosphate (cGMP) diffusing from granulosa cells [5, 6]. Cyclic GMP is synthetized by natriuretic peptide receptor 2 (NPR2) [7], a key membrane-bound guanylyl cyclase expressed in follicular cells [8]. The concentration of cGMP in the preovulatory follicle decreases in response to luteinizing hormone (LH) [6].

In mice, LH activates two principal pathways that lead to a reduction in cGMP concentration in the oocyte. The first pathway consists of a reduction in cGMP transport from somatic follicular cells to the oocyte. This pathway is dependent on the kinase activity of the epidermal growth factor receptor (EGFR) and mitogen-activated protein kinases 3/1 (MAPK3/1) that are required for the phosphorylation of connexin 43 and gap junction closure [9–11]. In response to LH stimulus, EGF-like factors amphiregulin (AREG) and epiregulin (EREG) are expressed and released from mural granulosa cells in mice [12]. These peptides then trigger the resumption of meiosis and cumulus expansion in mice [12], pigs [13] and humans [14]. Both AREG and EREG are also able to cause a decrease in cGMP in cultured follicles [6]. However, the cGMP level may be reduced in somatic cells of the follicle even if the EGFR activity is inhibited or absent [10].

The second pathway of cGMP decrease in the mouse preovulatory follicle is based on the regulation of cGMP synthesis and hydrolysis. LH downregulates the expression of C-type natriuretic peptide precursor (*NPPC*) in granulosa cells and causes a rapid decline in C-type natriuretic peptide (CNP), a ligand and activator of the NPR2 [15]. In addition, LH rapidly decreases activity of the NPR2 without reducing the amount of NPR2 protein [8]. Next, the LH surge inhibits the production of estradiol that is essential for the promotion and maintenance of NPR2 expression [16]. Finally, the cGMP level in follicular cells may be efficiently reduced by an increased activity of the cGMP-hydrolyzing enzymes. In support of this assumption, the cGMP-specific phosphodiesterase activity (types 5/6) as well as the amount of PDE6C protein are increased in porcine oocyte-cumulus complexes (COCs) cultured in the presence of human chorionic gonadotropin and equine chorionic gonadotropin [17].

The hyaluronan synthase 2 (HAS2), prostaglandin-endoperoxide synthase 2 (PTGS2) and tumor necrosis factor alpha-induced protein 6 (TNFAIP6) have been shown to play key roles in the regulation of cumulus expansion [18–20]. The expression levels of these genes are increased by EGF-like peptides in the response to LH signaling in vivo and also after the addition of exogenous EGF-like peptides to cultured COCs in mice [21] and pigs [13]. MAPK3/1 activation in cumulus cells is absolutely essential for the expression of the cumulus expansion-related genes [21–24]. The most compelling evidence of the pivotal role of MAPK3/1 in the control of ovulation processes came from experiments with granulosa-cell-specific *MAPK3/1* double-knockout mice that failed to ovulate and the females were completely infertile [22]. In concert with these observations, the expression of *HAS2*, *TNFAIP6* or *PTGS2* was completely abolished in these MAPK3/1 null mice.

Several lines of evidence indicate that cGMP may block meiotic resumption and cumulus expansion via a MAPK3/1-dependent mechanism, since the activation of MAPK3/1 in cumulus cells is essential for these processes to occur [25]. An activator of a soluble guanylyl cyclase and nitric oxide (NO) donor, S-nitroso-N-acetyl-penicillamine (SNAP), was found to prevent LH-induced MAPK3/1 activation in cultured rat follicles and to inhibit oocyte meiotic resumption and cumulus expansion [26]. Correspondingly, a guanylyl cyclase inhibitor 1H-[1,2,4] oxadiazolo[4,3-a]quinoxalin-1-one mimicked the action of LH and induced MAPK3/1 phosphorylation [26]. However, no direct effect of cGMP on MAPK3/1 or an upstream signal transduction pathway was shown in that study. In porcine COCs, both atrial natriuretic peptide (ANP) and 8-bromo-cGMP (8-Br-cGMP) in doses of 0.5 and 1.0 mM moderately inhibited FSH-induced oocyte maturation and cumulus expansion and partially reduced the phosphorylation of MAPK3/1 in both oocytes and cumulus cells [27]. The analysis of MAPK3/1 activity was carried out at the end of the culture period in that study and the significance of this finding in terms of the regulation of early events of meiotic resumption is not clear. In a recent paper, Santiquet et al. [28] reported no effect of ANP itself on cGMP production and FSH-induced meiotic resumption of pig cumulus-enclosed oocytes and the cGMP analogues only exhibited an inhibitory effect at a concentration of 5 mM, well above the range used in all previously published studies. The effect of CNP on the maturation of intact pig COCs is not clear either. So far, it has been shown that CNP inhibits the meiotic resumption of pig cumulus-enclosed oocytes pre-cultured for 22 h in dibutyryl cAMP [29]. Santiquet et al. [28] reported an inhibition of meiotic resumption in pig cumulus-enclosed oocytes treated with 10 μM CNP whereas nM concentrations are effective in mice [15]. We assumed that further studies would be needed to clarify the role of cGMP in regulation of pig oocyte meiotic resumption and cumulus expansion.

The aim of this study was to determine whether the resumption of oocyte meiosis and expansion of cumulus cells in isolated pig COCs can be blocked by high levels of cGMP and whether this effect is mediated by the cGMP-dependent inhibition of MAPK3/1 in cumulus cells.

## Methods

The study was conducted in the laboratories of IAPG in Libechov in 2013 and 2014. All the procedures with animals were performed according to good veterinary practice for animal welfare according to the relevant Czech laws (No. 246/1992 and 419/2012).

### Culture media and reagents

All chemicals were purchased from Sigma (Prague, Czech Republic) unless otherwise specified.

### Collection of cumulus-oocyte complexes

Ovaries were obtained from premature crossbred gilts (Landrace and Large White), 6–8 months old and 90–120 kg in weight, originating from two commercial breeding farms. The animals were slaughtered at a local abattoir in the city of Mimon, their ovaries excised and transported to the laboratory in a thermo-flask at 38°C. The contents of medium-size antral follicles about 3–5 mm in diameter were aspirated with a syringe connected to 20 G needle, pooled in a test-tube and allowed to sediment for 10 min. The sediment was washed twice with PBS, placed in a Petri dish and the COCs were collected by a pipette. Only COCs surrounded by compact multi-layered cumulus were selected for experiments.

### Culture of cumulus-oocyte complexes in vitro

The COCs were cultured in M-199 medium (Gibco, Life Technologies, Rockville, USA) supplemented with 0.91 mM sodium pyruvate, 0.57 mM cysteine, 5.5 mM Hepes, antibiotics and fetal calf serum (5%). Groups of 25–30 COCs were cultured in four-well dishes (Nunclon, Roskilde, Denmark) in 0.5 ml of culture medium at 38.5°C in a humidified atmosphere of 5% CO_2_. To stimulate the expansion of cumulus cells and oocyte maturation, the culture medium was supplemented with follicle-stimulating hormone (FSH) from sheep pituitary. Denuded oocytes were prepared mechanically by a repeated pipetting of intact COCs through a fine bore pipette.

### Assessment of oocyte maturation and cumulus expansion

To assess their nuclear maturation, oocytes were stripped of cumulus cells by vortexing, mounted on slides and fixed in an acetic acid-ethanol solution (1:3) for 48 h. Oocytes were then stained with 1% orcein and observed with a light microscope. Oocytes were scored for germinal vesicle (GV), germinal vesicle breakdown (GVBD; comprising mostly oocytes at metaphase I stage and few oocytes at late diakinesis, anaphase I or telophase I) and for metaphase II stage (MII). The degree of cumulus expansion was assessed 24 h after the onset of culture using a subjective scoring method [30]. Briefly, no response was scored as 0, minimum observable response, the cells in outermost layer of the cumulus becoming round and glistening as 1, the expansion of outer COCs layers as 2, the expansion of all COCs layers apart from *corona radiata* as 3 and the expansion of all COCs layers as 4. COCs exhibiting degree 3 and 4 were considered to be expanding.

### Measurement of cGMP concentration in COCs

Groups of 30 control, cGMP analogue-, or CNP-treated COCs were cultured for various time intervals, carefully washed in three large drops of PBS, and then lysed in 200 μl of 0.1 M HCl supplemented with Triton X-100 (0.01%) at room temperature for 30 min. During this period, the samples were briefly and vigorously vortexed every 10 min and centrifuged at the end of the lysis period at 10 000 g for 1 min. The supernatant was transferred into a new tube and stored at -20°C until use. The cGMP concentrations in the samples were determined with a cGMP complete ELISA kit (Enzo Life Sciences, Famingdale, NY, USA) according to the manufacturer’s instructions. Briefly, 100 μl of cGMP standards or samples were added to wells coated with affinity-purified antibody (goat-anti rabbit IgG). A blue solution of cGMP conjugated to alkaline phosphatase (ALP) was then added, followed by a yellow solution of rabbit polyclonal antibody to cGMP. The plate was incubated for 2 h on a shaker at room temperature to promote the immune reaction of endogenous and ALP-conjugated exogenous cGMP with the antibody and binding of the complex to the affinity antibody. The wells were emptied after incubation and extensively washed three times with the supplied wash-buffer to leave only bound cGMP in the wells. A total of 200 μl of p-nitrophenyl phosphate solution, an alkaline phosphatase substrate, was then added into each well and the plate was incubated for 1 h at room temperature in the dark without shaking. The reaction was stopped by trisodium phosphate solution and the amount of signal, proportional to the amount of cGMP in the sample, was measured with an ELISA plate reader (Synergy HT, BioTek, Winooski, VT, USA) at 405 nm. The cGMP concentrations in the samples were calculated from a standard curve and are expressed as fmol cGMP/COC.

### Real-time reverse transcription-polymerase chain reaction

The total RNA from 30 COCs cultured for various periods of time was isolated using RNeasy Mini Kit (Qiagen, Hilden, Germany) following the manufacturer’s instructions. Real-time RT-PCR was carried out in a RotorGene 3000 cycler (Corbett Research, Sydney, Australia) using One-Step RT-PCR Kit (Qiagen) with gene specific primers shown in Table 1. The 20 μl total reaction volume contained QIAGEN OneStep RT-PCR Buffer (1×), dNTP Mix (400 μM final concentration of each), reverse and forward primers (both 400 nM final concentration), SybrGreenI (0.4 μl of 1:1000 stock solution, Molecular Probes, Eugene, OR, USA), RNasine inhibitor (5 IU, Promega, Madison, WI, USA), QIAGEN OneStep RT-PCR Enzyme Mix (0.8 μl) and template RNA (2 μl). The reaction conditions were as follows: reverse transcription at 50°C for 30 min, pre-denaturation at 95°C for 15 min, followed by various numbers of PCR cycles, each of which consisted of denaturation at 95°C for 30 sec, annealing at a temperature specific for each pair of primers (shown in Table 1) for 20 sec, extension at 72°C for 20 sec, and a final extension step at 72°C for 5 min. The specificity of the PCR product was verified by melting analysis. The relative concentrations of the templates in the various samples were determined using comparative analysis software (Corbett Research). The results for individual target genes were normalized according to the relative concentration of the internal standard, *ACTB*.

**Table 1:** **Primers used for real-time RT-PCR**

Gene transcript	Primers	Amplicon length (bp)	T _an_(°C)	GenBank accession number
*ACTB*	F: 5′ – GAG AAG CTC TGC TAC GTC G – 3′	264	58	XM_3357928
	R: 5′ – CCA GAC AGC ACC GTG TTG G – 3′			
*HAS2*	F: 5′ – GAA GTC ATG GGC AGG GAC AAT TC – 3′	407	54	NM_214053
	R: 5′ – TGG CAG GCC CTT TCT ATG TTA – 3′			
*NPPC*	F: 5′– AGA AGG GCG ACA AGA CTC CT – 3′	182	*59*	NM_001008482
	R: 5′– GAA GCA GCC CTT GGA CAA AC – 3			
*NPR2*	F: 5′– TGC TTT GAT GCC ATA ATT GA – 3′	137	50	NM_001244322
	R: 5′–AAT GCT AGG GCC ATA CGA G – 3′			
*PTGS2*	F: 5′ – TCG ACC AGA GCA GAG AGA TGA GAT – 3′	260	55	NM_214321
	R: 5′ – ACC ATA GAG CGC TTC TAA CTC TGC – 3′			
*TNFAIP6*	F: 5′ – CAG AAG ACA TCA TTA GTA – 3′	150	54	NM_001159607
	R: 5′ – CAG TAG AAG TAG TAG TTG – 3′			

T_an_ - annealing temperature.

### Immunoblotting

At the selected culture time, COCs were washed in PBS and solubilized in Laemmli buffer containing 2% sodium dodecyl sulphate (SDS) and 5% 2-mercaptoethanol. Samples were boiled at 100°C for 3 min and stored frozen at -20°C. Subsequently, proteins were separated in 10% acrylamide/SDS gels and transferred to Immobilon-P membranes (Millipore, Bedford, MA, USA). Membranes were blocked in 5% low-fat dry milk in Tris-buffered saline (TBS) with 0.5% Tween 20 for 2 h at room temperature and then incubated with a primary antibody diluted 1:1000 in 5% BSA in TBS-Tween, at 4°C overnight. The primary antibodies were p-ERK and ERK1 (detecting MAPK3/1), both from Santa Cruz Biotechnology (Santa Cruz, CA, USA). The secondary antibodies (Amersham ECL anti-mouse or anti-rabbit IgG, GE Healthcare, Little Chalfont, UK) were diluted 1:5000 in 2% BSA in TBS-Tween. The membranes were incubated with the secondary antibody for 1 h at room temperature and washed intensively in TBS-Tween. The immune reaction was detected by enhanced chemiluminescence (Pierce, Rockford, IL, USA) according to the manufacturer’s instructions. The intensity of the specific bands on the blots was analyzed by scanning densitometry using the free software Image J Version 1.29 (National Institute of Mental Health, Bethesda MD, USA).

### Experimental design

*Experiment I* examined the expression of *NPR2* and *NPPC* during the culture of COCs in vitro (0–20 h).

*Experiment II* assessed the effect of cGMP analogues on intracellular cGMP concentration in the COCs. For this purpose, we used two synthetic cGMP analogues, 8-chlorophenylthio-cGMP (8-CPT-cGMP) and 8-Br-cGMP (both 0.1–1.0 mM) to control the concentrations of cGMP in the pig COCs. In all cases, the cGMP concentration in COCs was measured with a cGMP complete ELISA kit that recognized the endogenously produced cGMP and, as we found in preliminary experiments, also the synthetic cGMP analogues.

In *Experiment III*, the dynamics of cGMP production upon the stimulation of the COCs with CNP was monitored during the culture in vitro (0–20 h) and the effect of FSH on the CNP-stimulated production of cGMP was evaluated. For this purpose, the COCs were pre-cultured in CNP-supplemented medium for 1 h and then FSH was added to the cultured COCs. Alternatively, CNP and FSH were added simultaneously at the beginning of culture.

In *Experiment IV*, we assessed the effect of 8-CPT-cGMP, 8-Br-cGMP and CNP on the maturation of oocytes, the expansion of cumulus cells and expression of cumulus expansion- related genes. The COCs were exposed to the cGMP analogues or CNP for 1 h before the addition of FSH. The time of FSH addition was then taken to be time 0 h.

Finally, in *Experiment V*, we investigated the effect of 8-CPT-cGMP and CNP on the FSH-induced activation of MAPK3/1 in cumulus cells.

### Statistical analysis

The statistical analyses were performed with the software GraphPad Prism 5.0 (La Jolla, CA, USA). Each experiment was performed in at least 3 replicates. The differences in the percentages of maturing oocytes, the concentration of cGMP in COCs, the densitometrical quantifications of MAPK3/1 and the quantifications of RT-PCR results were compared by the analysis of variance (ANOVA) followed by Tukey’s post-test as in previous papers published by our and other laboratories [2, 6, 8, 13, 21, 23, 24, 28]. The temporal differences in maturation and the differences in cGMP concentration between control and CNP treated COCs or FSH and FSH + CNP treated COCs were compared by unpaired *t* test. The Kolmorogov-Smirnov test was used to examine normal distribution of all data. A level of P < 0.05 was considered significant. Error bars indicate the standard error of the mean (SEM).

## Results

### Expression of *NPPC*and *NPR2*in cultured COCs

**Figure 1:**
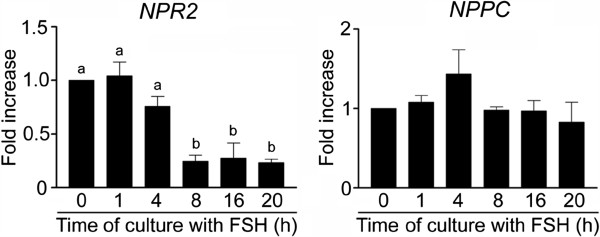
**Expression of** ***NPR2*** **and** ***NPPC*** **in COCs cultured in vitro in FSH-supplemented medium.** The relative abundance of specific gene mRNA was assessed in COCs cultured with FSH (1 IU/ml) for the indicated periods of time by a real-time RT-PCR and is expressed in arbitrary units as fold increases in the specific gene/*ACTB* ratio over the level found in control COCs at time 0 h. The different superscripts above the columns indicate significant differences (P < 0.05).

This experiment confirmed the expression of both genes involved in the production of cGMP in freshly isolated pig COCs. However, the pattern of *NPR2* expression during the culture in vitro with FSH was markedly different from that of *NPPC*. We found that the expression of *NPR2* in the FSH-stimulated COCs significantly (P < 0.05) decreased after 4 h of culture and remained low until the end of the experiment at 20 h. In contrast, the expression of the NPR2 ligand, *NPPC* remained stable (P > 0.05) over the whole of the culture period (Figure 1).

### Effect of CNP and FSH on the concentrations of cGMP in cultured COCs

**Figure 2:**
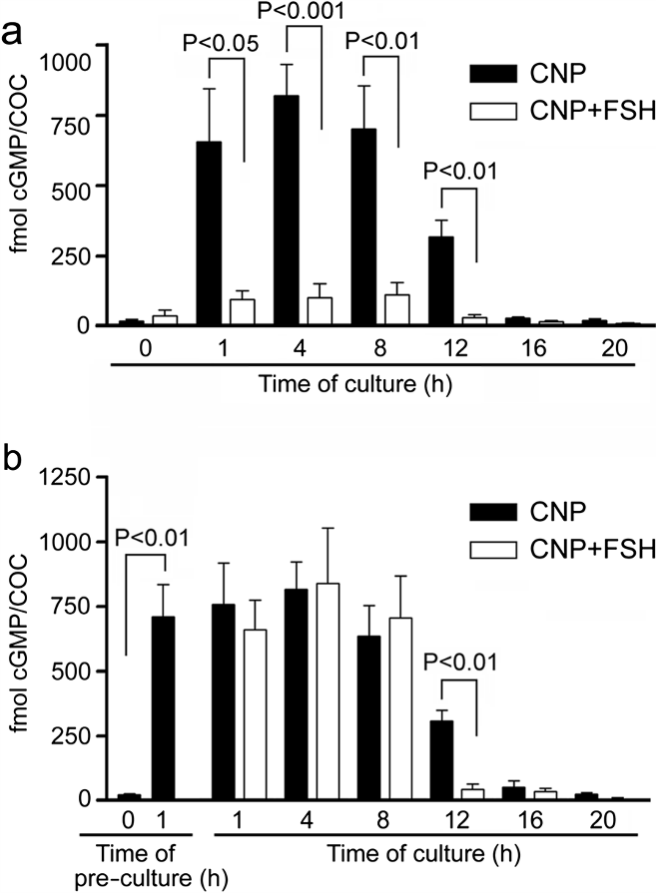
**Effect of CNP and FSH on cGMP concentration in cultured COCs. (a)** COCs were stimulated by simultaneous addition of CNP (100 nM) and FSH (1 IU/ml) in culture medium. **(b)** COCs were pre-treated with CNP for 1 h before FSH addition at the time 0 h. The significant differences between CNP and CNP + FSH groups within the same interval of culture are shown above the columns.

Consistent with our finding that both the *NPR2* and *NPPC* genes are expressed in intact COCs, CNP (100 nM) was found to be highly capable of stimulating the production of cGMP in the cultured COCs. The concentration of cGMP in COCs increased approximately 15-fold during the first h of culture (P < 0.01) and peaked at 4 h of culture, exhibiting an approximately 30-fold increase over the base level (P < 0.001) (Figure 2a). The CNP-stimulated concentrations of cGMP remained high (>600 fmol/COC) during the first 8 h of culture, dropped to approximately 300 fmol/COC at 12 h and returned to a base level at 16 h. FSH suppressed the dramatic CNP-induced increase in cGMP concentration if added simultaneously with CNP (Figure 2a). However, the high level of cGMP induced by 1 h pre-culture of the COCs with CNP was not reduced by FSH for 8 h of culture, but it was significantly (P < 0.01) lowered at 12 h of culture (Figure 2b).

### cGMP analogues increase cGMP concentration in cultured COCs

**Figure 3:**
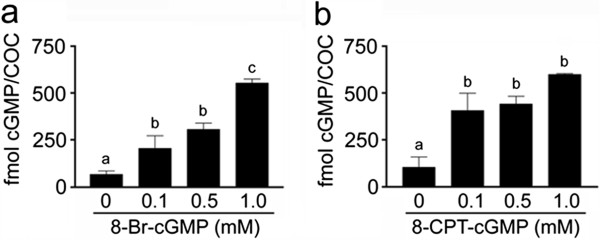
**Effect of cGMP analogues on cGMP concentration in cultured COCs. (a)** COCs were cultured in medium supplemented with various concentrations of 8-Br-cGMP or **(b)** 8-CPT-cGMP for 4 h. The different superscripts above the columns indicate significant differences (P < 0.05).

Both 8-Br-cGMP and 8-CPT-cGMP (0.1–1.0 mM) significantly (P < 0.05) increased the concentration of a measurable intracellular cGMP in the COCs at 4 h of culture to levels comparable with those seen in the CNP-treated COCs (Figure 3).

### cGMP analogues and CNP affect maturation of porcine oocytes, but not expansion of cumulus cells

**Figure 4:**
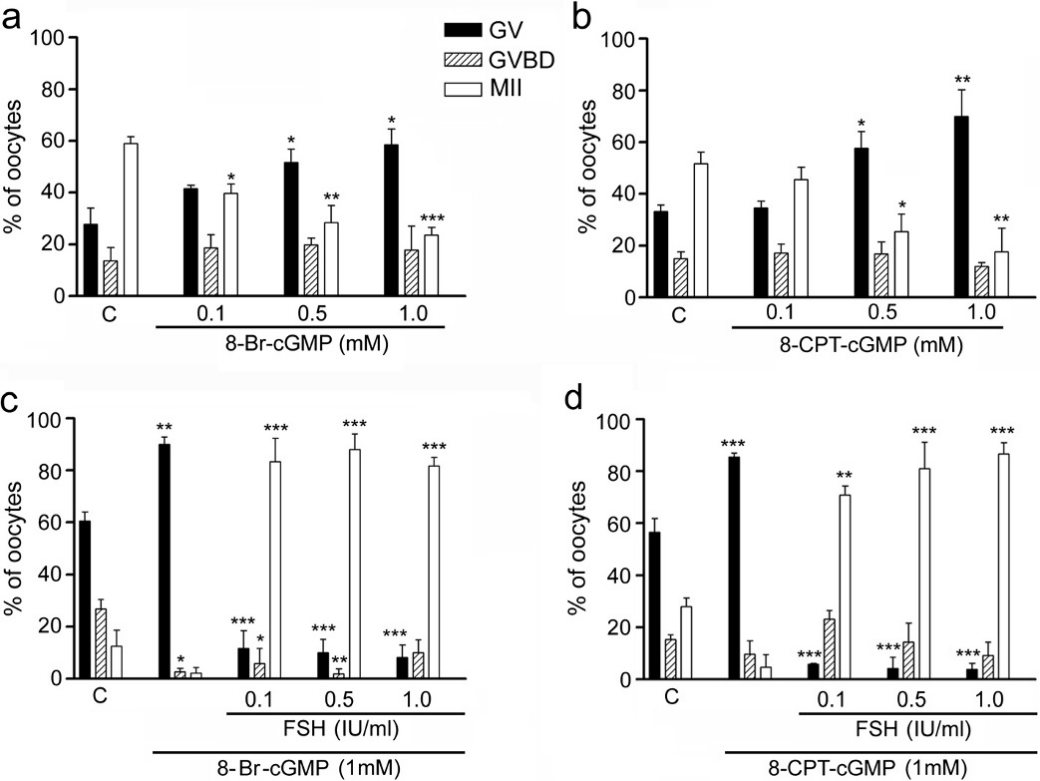
**Effect of cGMP analogues on maturation of pig denuded oocytes and COCs. (a)** Maturation of denuded oocytes cultured in medium supplemented with various concentrations of 8-Br-cGMP or **(b)** 8-CPT-cGMP for 44 h. **(c)** Effect of FSH on maturation of COCs in medium supplemented with 1 mM 8-Br-cGMP or **(d)** 1 mM 8-CPT-cGMP for 44 h. C, control oocytes or COCs cultured in medium without FSH and cGMP analogues; GV, germinal vesicle; GVBD, germinal vesicle breakdown; MII, metaphase II. Asterisks above the column indicate significant differences from the corresponding value in the control group. *P < 0.05; **P < 0.01; ***P < 0.001. Approximately 75 denuded oocytes or COCs were included in each group.

In vitro, exogenous 8-Br-cGMP and 8-CPT-cGMP (0.5–1.0 mM) efficiently blocked the maturation of denuded oocytes (Figure 4, a and b) as well as the spontaneous maturation of COCs (1 mM) (Figure 4, c and d), since significantly more oocytes remained at the GV stage compared to the control group of denuded oocytes or COCs cultured in the medium without analogues. The inhibitory effect of the cGMP analogues on COCs maturation was completely reversed by the addition of FSH into the culture medium in a dose as low as 0.1 IU/ml (Figure 4, c and d).

**Figure 5:**
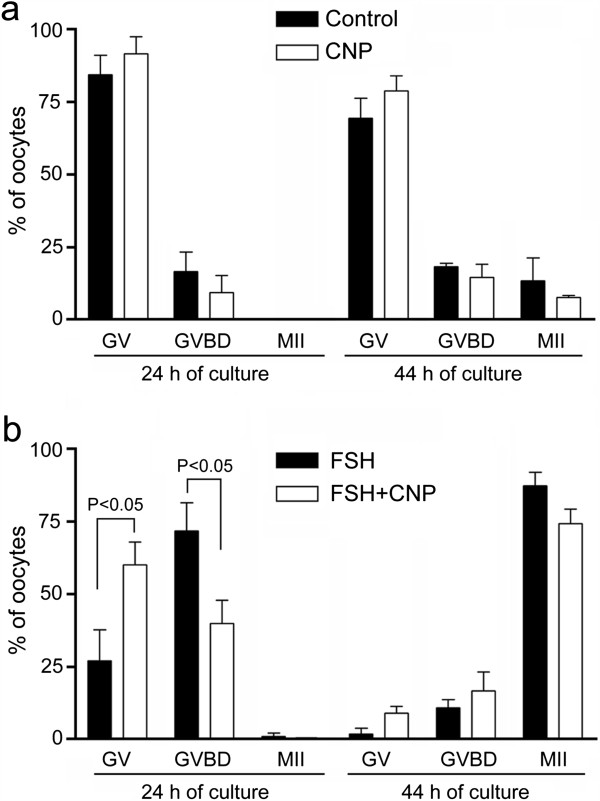
**Effect of CNP on spontaneous and FSH-induced maturation of pig cumulus-enclosed oocytes. (a)** Effect of CNP (100 nM) on spontaneous maturation of oocytes. **(b)** Effect of CNP on maturation of oocytes induced by FSH (1 IU/ml). GV, germinal vesicle; GVBD, germinal vesicle breakdown; MII, metaphase II. Significant differences were only found between FSH and FSH + CNP groups after 24 h of culture. Approximately 75 COCs were included in each group.

The effect of CNP was only tested on COCs, since NPR2 receptors are present on the surface of cumulus cells, but not on the oocyte itself [7, 31]. The CNP neither affected the spontaneous maturation of the COCs (Figure 5a) nor the FSH-induced maturation of COCs assessed after 44 h of culture (Figure 5b). However, we found that CNP significantly (P < 0.05) decreased the percentage of COCs that underwent GVBD after 24 h of culture (Figure 5b). This indicates that the CNP-induced increase in cGMP concentration delayed the FSH-induced meiotic resumption of pig oocytes in vitro.

**Figure 6:**
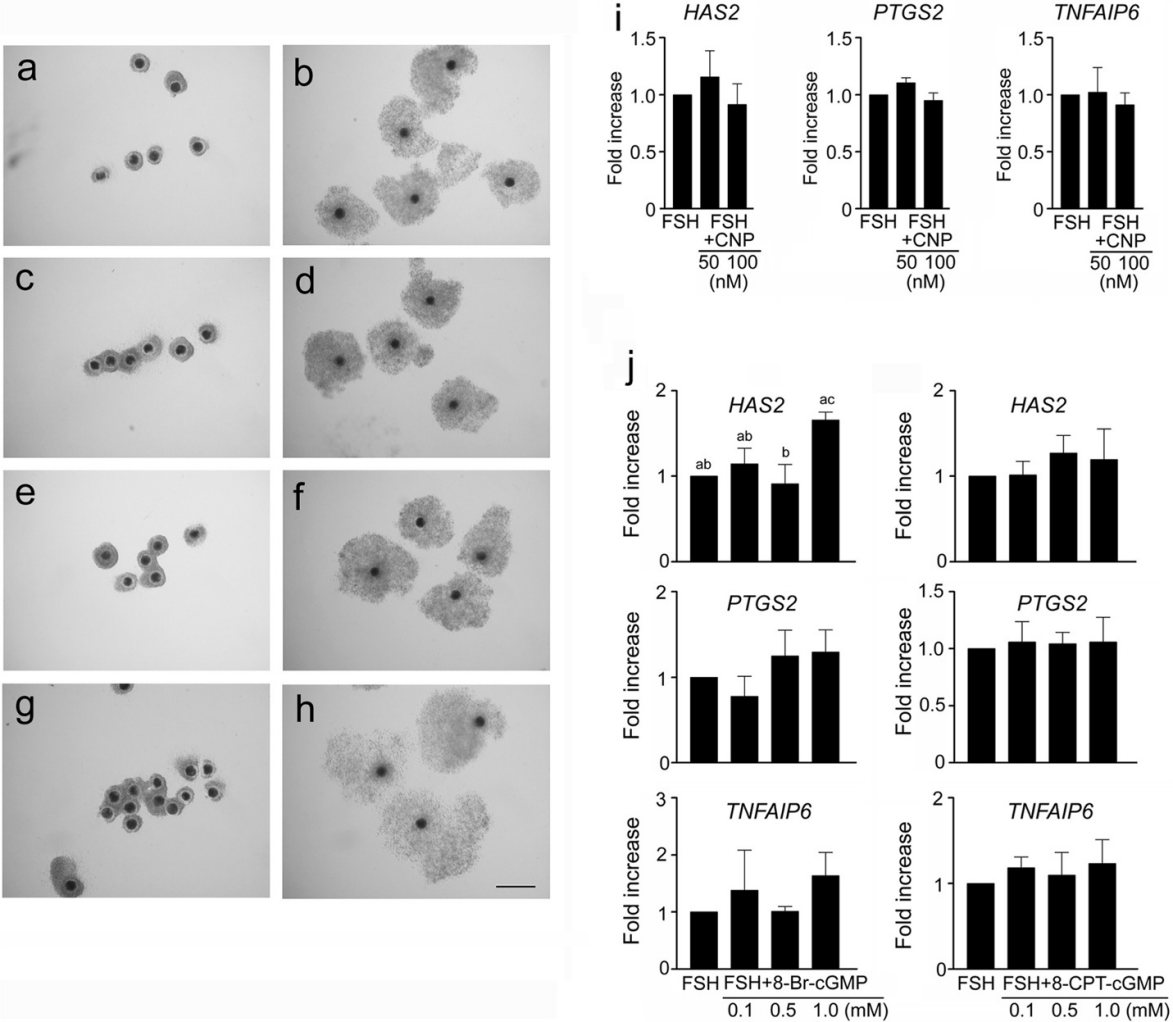
**Effect of FSH, cGMP analogues and CNP on cumulus expansion and expression of cumulus expansion-related genes in cultured COCs. (a)** COCs cultured in control medium. **(b)** COCs cultured in medium supplemented with FSH (1 IU/ml). **(c)** COCs cultured in medium with 8-CPT-cGMP (1 mM). **(d)** COCs cultured in medium with FSH and 8-CPT-cGMP. **(e)** COCs cultured in medium with 8-Br-cGMP (1 mM). **(f)** COCs cultured in medium with FSH and 8-Br-cGMP. **(g)** COCs cultured in medium with CNP (100 nM). **(h)** COCs cultured in medium with FSH and CNP. Bar = 400 μm. **(i)** Effect of CNP and **(j)** cGMP analogues on FSH-induced expression of cumulus expansion-related genes. The relative abundance of specific gene mRNA was assessed by real-time RT-PCR and is expressed in arbitrary units as fold increases in the specific gene/*ACTB* ratio over the level found in COCs stimulated with FSH (1 IU/ml). The different superscripts above the columns indicate significant differences (P < 0.05).

The COCs did not undergo expansion in the control medium (Figure 6a). In contrast, all COCs underwent expansion in the FSH-supplemented medium (Figure 6b). Both cGMP analogues as well as the CNP neither stimulated expansion of the COCs (Figure 6, c, e and g) nor decreased the degree of FSH-stimulated cumulus cell expansion since virtually all COCs (*n* = 75 in each group) exhibited an expansion of all layers of cumulus cells except the *corona radiata* cells (Figure 6, d, f and h).

### CNP and cGMP analogues did not downregulate the expression of cumulus expansion-related genes

The expression of *HAS2*, *PTGS2* and *TNFAIP6* in COCs was enhanced approximately 17, 20 and 1000-fold by FSH, respectively, at 4 h of culture. The FSH-induced expression of each specific gene was not significantly (P > 0.05) affected by CNP (Figure 6i) or by the cGMP analogues (Figure 6j).

### MAPK3/1 activation was not affected by high concentration of cGMP

**Figure 7:**
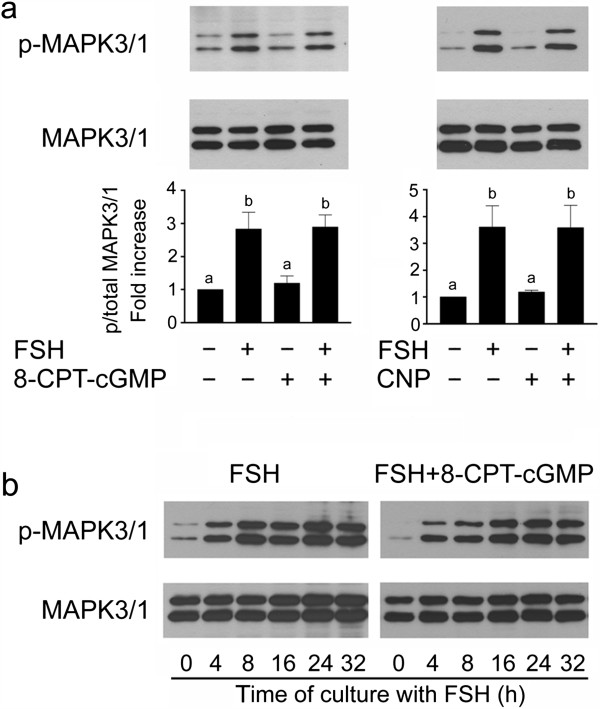
**Effect of FSH (1 IU/ml), CNP (100 nM) and 8-CPT-cGMP (1 mM) on activation of MAPK3/1 in cultured COCs. (a)** The images show a representative result of immunoblotting of phosphorylated MAPK3/1 (top panels) and total MAPK3/1 (bottom panels) in samples of 25 COCs cultured for 4 h. The graphs represent quantification of the activated MAPK3/1 in COCs by densitometry. The results are shown as proportions of the phosphorylated and total MAPK3/1 and expressed in arbitrary units as a fold strength increase over the proportion found in COCs cultured in control medium. The different superscripts above the columns indicate significant differences (P < 0.05). **(b)** Time course of MAPK3/1 activation in COCs cultured in medium supplemented with FSH or FSH + 8-CPT-cGMP.

By immunoblotting with phospho-specific antibodies, we detected a significant (P < 0.05) increase in MAPK3/1 phosphorylation in FSH-stimulated COCs at 4 h of culture. Neither CNP nor 8-CPT-cGMP affected the basal level of MAPK3/1 phosphorylation and did not reduce MAPK3/1 phosphorylation induced by FSH (P > 0.05) (Figure 7a). The high level of MAPK 3/1phosphorylation persisted in FSH-stimulated COCs for at least 32 h of culture, when the cumuli displayed maximum expansion. The cGMP analogue (8-CPT-cGMP, 1 mM) did not change the pattern of MAPK 3/1 phosphorylation during the culture of COCs in vitro (Figure 7b).

## Discussion

The data from our study show, for the first time in a non-rodent species, the pattern of *NPPC* and *NPR2* expression in COCs cultured in vitro. Both *NPPC* and *NPR2* are expressed in the intact COCs, but only the *NPR2* seems to be downregulated during the culture in vitro with FSH. The *NPR2* downregulation observed in our study may thus be involved in the decrease in cGMP contents in cumulus cells and consequently in the oocyte. Therefore, this step may participate in the regulation of meiotic resumption in cultured pig oocytes. The downregulation of *NPR2* in the COCs occurred between 4 and 8 h of culture, which further supports the possible role of this gene in the regulation of meiotic resumption since all essential morphological events associated with the transition of pig oocytes from GV to metaphase I, such as chromosome condensation and nuclear membrane breakdown, occur after 8 h of culture in vitro [32]. In principle, two mechanisms leading to a decrease in cGMP production in the granulosa and cumulus cells of preovulatory follicles have been described. The first is a LH-induced decrease in the expression of *NPPC* leading to a significant drop in ovarian CNP content 2 h after hCG injection [15]. The second is a LH-inhibited activity of the NPR2 that occurs in granulosa cells in a post-translational manner within 10–20 min [8, 33], followed by a reduced activity of NPR2 in cumulus cells that occurs within 2 h [8]. It follows that both mechanisms may play a role in the promotion of meiotic resumption in the mouse, since GVBD was found in that study at 3 h after hCG injection [8]. In pig COCs cultured in vitro, only a FSH-induced decrease in *NPR2* expression appears to be involved in the regulation of meiotic resumption, as proposed in our study.

The reduction of cGMP transport from somatic follicular cells to the oocyte is another mechanism contributing to the release of oocytes from meiotic arrest. In mice and rats, gonadotropins may induce rapid gap junction closure via activation of the MAPK3/1-dependent pathway [34] and thus avoid the diffusion of cGMP into the oocyte. However, the role of gap junction permeability in meiotic resumption in pigs is still not clear. Surprisingly, gap junction permeability is increased in a gonadotropin-independent manner during the first hours of COCs culture and a gonadotropin-dependent closure of gap junctions occurs before or simultaneously with GVBD [35, 36]. Thus, it appears that the deregulation of gap junctions may play a role in the initiation of meiotic resumption in pig COCs. However, it does not occur immediately after gonadotropin stimulation, as proposed in rodent models, but is postponed beyond 8 h of culture, reflecting the extended period of time required for GVBD in pig oocytes. Taken together with our data, it appears that both downregulation of *NPR2* and a reduction in gap junction communication may contribute to the decline in cGMP in cumulus cells and oocytes, respectively, and to meiotic resumption of pig oocytes in vitro.

In agreement with the expression of *NPPC* and *NPR2* found in our study, the COCs were highly responsive to exogenous CNP in terms of cGMP production. Stimulation of the COCs with 100 nM CNP resulted in an approximately 30-fold increase in the basal level of cGMP in 4 h. This is in agreement with data reported for the mouse COCs, where 10–100 nM of CNP induced several-fold increases in cGMP concentration, associated with the inhibition of oocyte meiotic resumption [15, 37]. However, the concentration as high as 10 μM of CNP was required for inhibition of FSH-induced meiotic resumption in pig oocytes, which was associated with a threefold increase in cGMP concentration in the COCs after 24 h of culture [29]. We found that nM concentrations of CNP caused up to 30-fold increase in cGMP levels in COCs during the first 8 h of culture and that the cGMP levels returned to a base after 16–20 h of culture. Next, we showed that FSH prevented the rise in cGMP from the basal concentrations, if added simultaneously with CNP. A possible explanation of this phenomenon is that FSH caused a rapid desensitization of NPR2 via protein kinase C and calcium-dependent signaling and reduced thus NPR2 affinity for CNP as observed in 293 T-NPRB cells [38]. Finally, we showed that FSH did not reduce the high cGMP levels generated in CNP-pre-cultured COCs during the first 8 h of culture, but it accelerated the drop in cGMP to a base level thereafter. In pigs, cGMP-specific phosphodiesterases 5A and 6C were permanently upregulated beyond 12 h of culture COCs in the response to gonadotropin stimulation [17]. These cGMP-specific phosphodiesterases may also be responsible for the FSH-accelerated decrease in cGMP concentration observed in our study.

In this study, we also focused on the effect of cGMP analogues on the maturation of porcine denuded and cumulus-enclosed oocytes. The analogues dramatically increased the intracellular concentration of measurable cGMP in the COCs and significantly decreased the percentage of oocytes resuming meiosis. The cGMP analogues may inhibit meiotic resumption via the same mechanism as the endogenously produced cGMP, i.e. by inhibiting the oocyte PDE3A [5, 6]. However, we cannot preclude alternative mechanism(s). Zhang et al. [27, 39] reported that protein kinase G-specific inhibitor KT5823 reversed the inhibitory effects of ANP and 8-Br-cGMP on porcine COC meiotic maturation, suggesting the possible involvement of this kinase. In this study, the inhibitory effect of cGMP analogues on maturation of cumulus enclosed oocytes was completely reversed by the addition of FSH into the medium at a concentration as low as 0.1 IU/ml (~10 ng/ml). This is in contrast to data published by Zhang et al. [27, 39], who reported a moderate inhibitory effect of 8-Br-cGMP on FSH-induced maturation of pig cumulus-enclosed oocytes. This discrepancy with our findings may be due to a different source and concentration of FSH, different culture medium (NCSC 37 vs. M199) and different protein supplement (follicular fluid vs. FCS).

The mechanism by which FSH overcomes the cGMP analogue-induced meiotic block is not completely clear. A possible explanation is that FSH caused a closure of gap junctions thereby limiting the supply of the analogue transported from cumulus cells to the oocyte. However, the direct influx of the analogues from the surrounding medium to the oocyte seems sufficient to block meiosis, as indicated by the inhibitory effect of cGMP analogues on meiotic resumption in denuded oocytes. Therefore, FSH may trigger an alternative pathway that can decrease or bypass the high intracellular concentration of cGMP and consequently also cAMP in oocytes. It has been shown that FSH can trigger meiotic resumption in mammalian oocytes inhibited at the GV stage by drugs increasing the intracellular concentration of cAMP, like hypoxanthine [40], dibutyryl cAMP [41] or PDE3A inhibitors [42] via a mechanism involving the protein kinase C and EGFR/MAPK3/1 pathways [41, 43, 44]. Moreover, the cGMP analogues are probably hydrolyzed in the COCs by cGMP-specific phosphodiesterases, the activity of which may be affected by FSH. In support of these assumptions, an inhibitor of phosphodiesterase 9A (PDE9A) enhanced the negative effect of 8-Br-cGMP on mouse oocyte meiotic resumption [45]. As mentioned previously, PDE5A and PDE6C, upregulated in response to gonadotropin, may also hydrolyze the cGMP analogues [17].

In cultured rat follicles, the NO donor SNAP was reported to prevent LH-induced activation of MAPK3/1 and downstream MAPK3/1-dependent events, such as disruption of the gap junctions, oocyte maturation and cumulus expansion [26]. Since the NO downstream effector is the guanylyl cyclase, the authors concluded that the actions of SNAP in the rat follicle are mediated by cGMP. In support of this conclusion, an inhibitor of soluble guanylyl cyclase induced oocyte maturation as well as cumulus expansion. The results of our study demonstrate that neither endogenous cGMP nor exogenous cGMP analogues interfere with the FSH-induced activation of MAPK3/1, expression of cumulus expansion-related genes and degree of expansion in porcine COCs cultured in vitro. However, we are not able to exclude the possibility that MAPK3/1 activation may be inhibited by NO itself or its donors like SNAP by a cGMP-independent mechanism. NO can block the activity of constitutively active RAS and RAF1 proteins [46] as well as the activity of EGFR [47]. The S-nitrosylation of C-terminal cysteine residues may even increase the activity of RAS [48], probably due to an artificially high concentration of NO [45]. These observations may explain the dual effect of SNAP and other NO-donors on the meiotic maturation reported by some studies [49, 50]. NO-synthases were also detected in murine [51] and porcine [52, 53] oocytes but their role in meiotic maturation is still unclear.

Zhang et al. [27] reported that ANP and 8-Br-cGMP negatively regulates the FSH-induced cumulus expansion of porcine COC at 44 h of culture, due to the inhibition of MAPK3/1 signaling. We have shown in our study that 8-CPT-cGMP neither affected the onset of MAPK3/1 activation in the pig COCs at 4 h of culture nor the course of the kinase activation until 32 h of culture when the extent of cumulus expansion reached its maximum. A possible effect of 8-Br-cGMP on MAPK3/1 activation was not investigated. However, no significant effect of 8-Br-cGMP on the expression levels of cumulus expansion-related genes indicates that neither 8-CPT-cGMP nor 8-Br-cGMP can inhibit MAPK3/1 activation and cumulus expansion.

## Conclusions

In summary, the data of this study demonstrate that in vitro cultured pig COCs express key components of cGMP synthesis, *NPPC* and the *NPR2*, and produce a large amount of cGMP upon stimulation with CNP, which leads to a significant delay in FSH-induced oocyte meiotic resumption. The COCs also respond to exogenous cGMP analogues by inhibiting meiotic resumption, which can be reversed by stimulating the COCs with FSH. However, a high concentration of intracellular cGMP is not able to prevent FSH-induced activation of MAPK3/1 in cumulus cells, the expression of expansion-related genes and cumulus expansion. Thus, we conclude that the inhibitory effect of cGMP on meiotic resumption of pig cumulus-enclosed oocytes is not caused by the inhibition of MAPK3/1 activation in cumulus cell compartment. The inhibitory mechanism rather consists in the transport of cGMP to the oocyte and the maintenance of a high intracellular concentration of cAMP via the inactivation of PDE3A.

## Abbreviations

8-Br-cGMP: 8-bromo-cGMP
8-CPT-cGMP: 8-chlorophenylthio-cGMP
ALP: Alkaline phosphatase
ANP: Atrial natriuretic peptide
AREG: Amphiregulin
cAMP: Cyclic adenosine monophosphate
cGMP: Cyclic guanosinmonophosphate
CNP: C-type natriuretic peptide
COC: Cumulus-oocyte complex
EGFR: Epidermal growth factor receptor
EREG: Epiregulin
FSH: Follicle stimulating hormone
GV: Germinal vesicle
GVBD: Germinal vesicle breakdown
HAS2: Hyaluronan synthase 2
LH: Luteinizing hormone
MII: Metaphase II
MAPK3/1: Mitogen-activated protein kinase 3/1
NO: Nitric oxide
NPPC: C-type natriuretic peptide precursor
NPR2: Natriuretic peptide receptor 2
PDE3A: Phospodiesterase 3A
PTGS2: Prostaglandin-endoperoxide synthase 2
SNAP: S-nitroso-N-acetyl-penicillamine
TNFAIP6: Tumor necrosis factor alpha-induced protein 6.
